# Toward Green and Sustainable Zinc‐Ion Batteries: The Potential of Natural Solvent‐Based Electrolytes

**DOI:** 10.1002/smll.202411478

**Published:** 2025-01-21

**Authors:** Gulsah Yaman Uzunoglu, Recep Yuksel

**Affiliations:** ^1^ Department of Chemical Engineering Istanbul Health and Technology University (ISTUN) İstanbul 34445 Turkey; ^2^ Department of Chemistry Eskisehir Osmangazi University (ESOGU) Eskisehir 26040 Turkey; ^3^ Advanced Materials Technologies Application and Research Center (IMATEK) Eskisehir Osmangazi University (ESOGU) Eskisehir 26040 Turkey

**Keywords:** biomass electrolyte, dendrite suppression, natural solvent, sustainable battery, zinc‐ion battery

## Abstract

Zinc‐ion batteries (ZIBs) are emerged as a promising alternative for sustainable energy storage, offering advantages such as safety, low cost, and environmental friendliness. However, conventional aqueous electrolytes in ZIBs face significant challenges, including hydrogen evolution reaction (HER) and zinc dendrite formation, compromising their cycling stability and safety. These limitations necessitate innovative electrolyte solutions to enhance ZIB performance while maintaining sustainability. This review explores the potential of natural solvent‐based electrolytes derived from renewable and biodegradable resources. Natural deep eutectic solvents (DES), bio‐ionic liquids, and biomass‐derived organic compounds present unique advantages, including a wider electrochemical stability window, reduced HER activity, and controlled zinc deposition. Examples include DESs based on choline chloride (ChCl), glycerol‐based systems, and biomass‐derived solvents such as γ‐valerolactone (GVL) and aloe vera, demonstrating improved cycling stability and dendrite suppression. Despite their promise, challenges such as high viscosity, cost, and scalability remain critical barriers to commercialization. This review underscores the need for further research to optimize natural solvent formulations, enhance Zn anode compatibility, and integrate these systems into practical applications. By addressing these challenges, natural solvent‐based electrolytes can pave the way for safer, high‐performance, and environmentally sustainable ZIBs, particularly large‐scale energy storage systems.

## Graphical abstract

This perspective review aims to explore the potential of biomass‐derived materials to be utilized as natural solvents for zinc‐ion battery electrolytes, focusing on their sustainability, effectiveness, and ability to enhance battery performance.

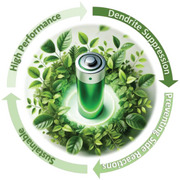

## 1 Introduction

### 1.1 Zinc‐Ion Batteries (ZIBs): Overview and Potential

Zinc‐ion batteries (ZIBs) have attracted significant attention due to their potential advantages in large‐scale energy storage systems. Compared to lithium‐ion batteries (LIBs), ZIBs offer benefits such as safety, low cost, and environmental friendliness, primarily due to the abundance of zinc, a non‐toxic and recyclable material.^[^ ^1^ ^]^ Additionally, using aqueous electrolytes in ZIBs provides a safer alternative to the flammable organic solvents used in LIBs. This makes ZIBs particularly appealing for applications such as grid storage, where long cycle life, cost‐effectiveness, and safety are critical factors. However, despite these advantages, aqueous ZIBs face critical challenges that limit their practical application. One of the primary issues is the hydrogen (H_2_) evolution reaction (HER), which occurs during charging, leading to H_2_ gas evolution and significantly jeopardizes the reversibility of Zn plating/stripping, resulting in low Coulombic efficiency (CE). Additionally, zinc dendrite formation, caused by the uneven deposition of zinc during cycling, can lead to short circuits and reduce battery lifespan. These issues necessitate the development of new electrolyte systems that can overcome the limitations of water‐based electrolytes while maintaining the advantages of Zn‐based batteries.^[^ ^2^, ^3^ ^]^

**Figure 1:**
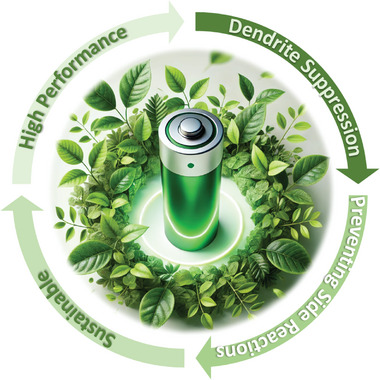
Characteristics of natural solvents in relation to designing electrolyte systems.

The aqueous electrolyte is one of the main reasons behind the limitations in ZIBs, and it is a good starting point for investigating possible solutions for the stability of ZIBs.^[^ ^4^ ^]^ Natural materials and their derivatives, obtained from renewable and sustainable resources, can be promising solutions to mitigate both HER activity and dendrite formation.^[^ ^3^ ^]^ The diverse structural configurations, improved surface chemistry, and significant eco‐friendliness of biomass materials position them as a viable option for developing high‐performance aqueous ZIBs (**Figure** **1**).^[^ ^5^ ^]^ Natural organic compounds, such as proteins,^[^ ^6^, ^7^ ^]^ amino acids,^[^ ^8^, ^9^ ^]^ saccharides,^[^ ^10^, ^11^ ^]^ and organic acids^[^ ^12^, ^13^ ^]^ have recently been shown to regulate the solvation structure of the electrolyte and to enhance the electrode/electrolyte interface of ZIBs. Natural materials provide non‐toxicity, high compatibility, biodegradability, and abundance, overlapping with the need for safe‐by‐design high‐performance ZIBs. This concept review aims to explore the potential of natural materials to be utilized as solvents for ZIB electrolytes, focusing on their sustainability, effectiveness, and ability to enhance battery performance.

## 2 Challenges of Aqueous Zinc‐Ion Batteries: Hydrogen Evolution and Zinc Dendrite Formation

The electrolyte, an inactive battery component, functions as a medium for ion transport between the anode and the cathode, enabling the electrochemical processes to occur during charge–discharge cycles. Electrolyte plays a pivotal role in determining battery performance.^[^ ^4^, ^14^ ^]^ Essential contributions of the electrolyte, including ionic conductivity, electrochemical stability window, viscosity, and compatibility with other battery components, significantly influence critical parameters such as capacity, power output, cycle life, safety, and stability.^[^ ^15^, ^16^ ^]^ Limited ionic conductivity in electrolytes can restrict charge and discharge rates, thereby diminishing power output. Similarly, a narrow electrochemical stability window constrains the battery's operating voltage range, ultimately reducing its capacity and energy density.^[^ ^14^ ^]^ For ZIBs, aqueous solutions of zinc salts are the preferred electrolytes due to their high ionic conductivity, ability to deliver high power density, and safety. However, the reduction potential of hydrogen (−0.41 V vs SHE) is higher than Zn (−0.76 V vs SHE), and the HER occurs when water molecules in the aqueous electrolyte are reduced to form H_2_ gas during the charging process. In aqueous electrolytes, H_2_ formation competes with the Zn deposition process, leading to a loss of efficiency and, in some cases, gas buildup within the battery, which can compromise safety. In addition, HER lowers the CE of the battery, reducing its overall performance. Water molecules in the aqueous electrolyte have two forms, which are free and bound, and the free water molecules form hydrogen bonding networks. Bound water molecules are coordinated with the Zn^2+^ ions in the electrolyte solution, and they have low electrochemical stability due to the charge transfer between Zn^2+^ and the water molecule. Recent studies reported that HER activity mainly originated from the free water molecules.^[^ ^17^, ^18^ ^]^ The phenomenon of self‐ionization in pure water is well recognized, wherein H_2_O dissociates to yield OH^−^ and H_3_O^+^ ions (2H_2_O ⇌ H_3_O^+^ + OH^−^). When zinc salts, such as zinc sulfate (ZnSO_4_) and zinc trifluoromethanesulfonate (Zn(OTf)_2_), are dissolved, the pH of the solution changes from neutral to slightly acidic in the aqueous solution of ZnSO_4_ or Zn(OTf)_2_. This drop in pH is attributed to the interaction between Zn^2+^ ions and water molecules, which facilitates the decomposition of water molecules. The electric field generated by Zn^2+^ applies a force on the water molecules, leading to electron transfer from coordinated H_2_O to the vacant orbitals of Zn^2+^. This interaction significantly weakens the O─H bonds in H_2_O, thereby promoting the HER. It is important to note that a limited amount of additives cannot completely inhibit the self‐ionization of water; thus, a substantial additive (co‐solvent) is required to foster strong interactions with water.^[^ ^19^ ^]^ The debates on the origin of water molecules resulting in the parasitic side reactions are still ongoing.^[^ ^5^, ^20^, ^21^ ^]^

Zn dendrite formation is another critical issue in ZIBs, and it presents a significant obstacle to achieving long‐term cycling stability. The uneven nature of the Zn deposition process leads to the formation of sharp, thin, hexagonal structures known as dendrites.^[^ ^22^ ^]^ These dendrites can grow and eventually puncture the separator, leading to internal short circuits and premature battery failure. Besides the mechanical damage caused by dendrites, they reduce the effective surface area of the anode, leading to an increase in the local current density and further promoting non‐uniform deposition. Several factors, including the electrolyte composition, Zn^2+^ concentration, current density, and temperature, influence the dendrite formation. Suppressing Zn dendrite formation is crucial for improving the long‐term stability and safety of ZIBs.^[^ ^23^ ^]^

Considering the aforementioned issues, such as HER, OER, and zinc dendrite formation, electrolyte composition plays a critical role in determining the performance and stability of ZIBs. One approach is using highly concentrated electrolytes to control water activity, reduce the availability of free water molecules, and extend the electrochemical stability window (ESW).^[^ ^24^ ^]^ However, this approach has limitations, including high viscosity, reduced ionic conductivity, and increased cost.^[^ ^19^ ^]^ Other strategies have also been employed to suppress dendrite formation and HER activity, including adjusting the electrolyte pH, adding inhibitors, or using protective coatings on the zinc anode. Unfortunately, these approaches often provide only partial solutions and may introduce additional complexities or costs.

**Figure 2:**
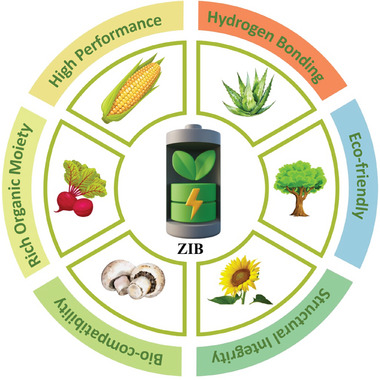
Advantages of bio‐based materials as natural solvents for ZIB electrolytes.

Given the challenges associated with aqueous electrolytes, natural solvent‐based systems have emerged as a promising alternative (**Figure** **2**). Within a few years, a steadily increasing number of studies have focused on modifying the electrolyte composition and exploring alternatives to decrease the water content.^[^ ^25^ ^]^ One promising approach is using natural solvent‐based electrolytes instead of traditional electrolyte systems.^[^ ^26^ ^]^ Natural solvents, typically having a higher ESW than water, such as organic compounds derived from biomass or deep eutectic solvents (DES), can potentially mitigate both HER and dendrite formation.^[^ ^27^, ^28^ ^]^ They help to control the zinc ion transport and deposition processes by altering the solvent environment, thereby suppressing the growth of dendrites. Natural solvent‐based systems can offer improved stability and performance by reducing water content and providing a more controlled environment for zinc ion transport and deposition.^[^ ^29^ ^]^ These solvents possess several key characteristics that make them suitable for use in ZIBs. A large ESW of natural solvent‐based electrolytes leads to higher CE and improved energy density.^[^ ^5^, ^30^ ^]^ One another major advantage of natural solvents is their sustainability. Derived from renewable biomass sources, these solvents offer a greener alternative to synthetic organic chemicals, reducing the environmental impact of battery production and recycling.^[^ ^5^ ^]^ The presence of hydrophilic functional groups in biomass, such as NH_2_, ─OH, ─CONH─, ─CONH_2_, and ─SO_3_H, contributes to a pronounced adsorption affinity for water and other polar solvent molecules. This strong interaction with salt anions not only enhances the solubility of salts but also promotes cation transport, resulting in a more even distribution of Zn^2+^ across the electrode surface, thereby mitigating the formation of Zn dendrites.^[^ ^24^, ^31^ ^]^

## 3 Classification of Natural Solvent‐Based Electrolytes

Natural solvents are organic compounds derived from renewable resources that offer more sustainable and green alternatives to conventional solvents used in energy storage devices.^[^ ^2^, ^13^, ^27^, ^32^ ^]^ Unlike petroleum‐based organic solvents, natural solvents provide a greener option due to their biodegradable nature, lower environmental footprint, and bio‐circularity.^[^ ^33^, ^34^, ^35^ ^]^ The controlled electrolyte environment provided by natural solvents helps to achieve a more uniform zinc‐ion flux, reducing the likelihood of dendrite growth. Natural solvent‐based electrolytes can be classified in four main groups, which are polyols (ethylene glycol, glycerol, and other polyols), bio‐ionic liquids, natural deep eutectic solvents (NADESs), and biomass‐derived organic solvents.

**Figure 3:**
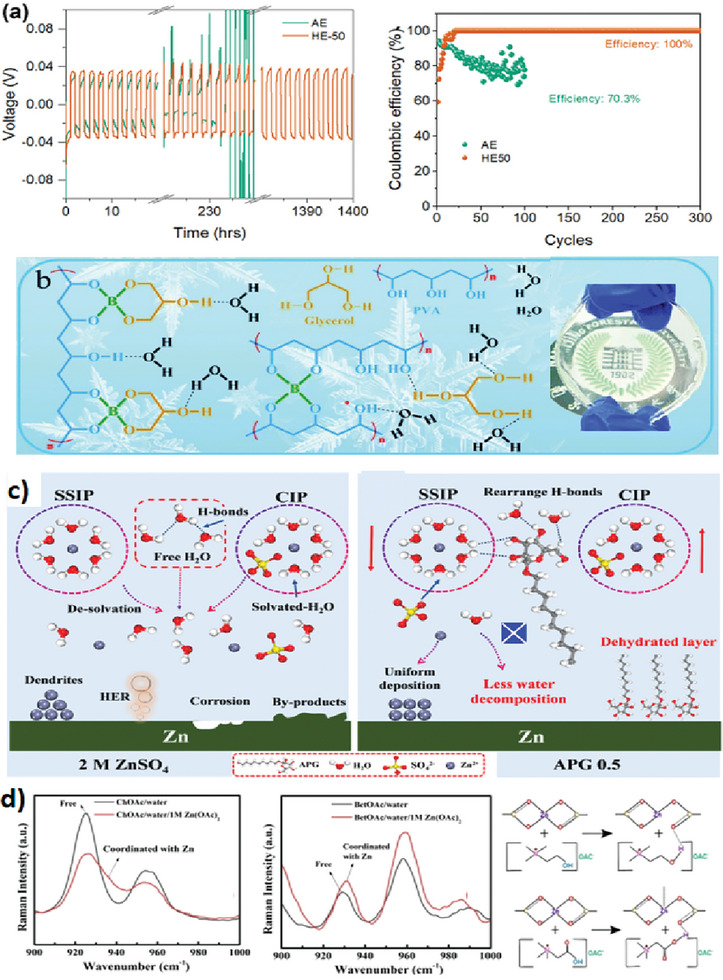
a) Zn stripping/plating of Zn/Zn symmetric cells in aqueous electrolyte (AE) and EG hybrid electrolyte (HE‐50) and Zn stripping/plating CE of Zn/Ti cells in AE and HE‐50. Reproduced from ref.[37] Copyright (2023), with permission from the publisher. b) Multi‐complexation of the integrated 3D network within a borax‐crosslinked polyvinyl alcohol (PVA)/glycerol gel electrolyte (PVA‐B‐G) and photographs of PVA‐B‐G film. Reproduced from ref.[38] Copyright (2020), with permission from the publisher. c) A schematic illustration for the zinc deposition process in aqueous ZnSO_4_ and APG/ZnSO_4_ electrolyte. Reproduced from ref.[40] Copyright (2024), with permission from the publisher. d) Raman spectra comparison of bio‐ILs; ChOAc and betaine acetate (BetOAc) in water, and the Zn(OAc)_2_ reaction with ChOAc and BetOAc. Reproduced from ref.[43] Copyright (2024), with permission from the publisher.

**Figure 4:**
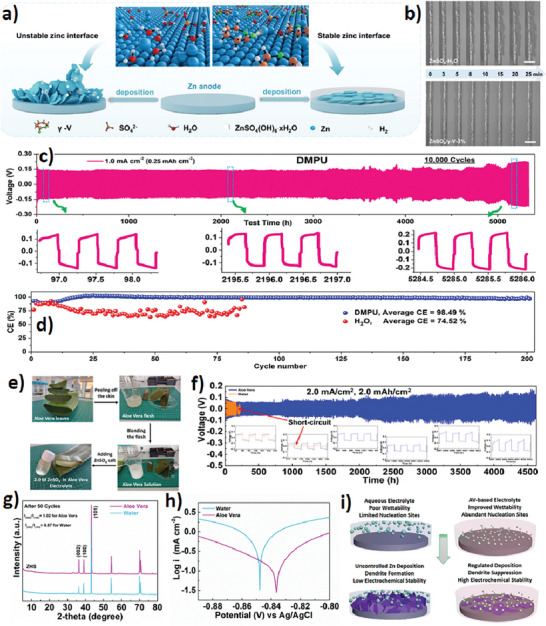
a) Schematic illustration for the Zn deposition process in the electrolyte without/with γ‐V additives. b) In situ optical microscope visualization of Zn plating process at 5 mA cm^−2^ in ZnSO_4_‐H_2_O and ZnSO_4_/GVL‐3% electrolytes. Reproduced from ref.[53] Copyright (2024), with permission from the publisher. c) Zn/Zn cell cycle response under the condition of 1.0 mA cm^−2^ (0.25 mAh cm^−2^) in DMPU electrolyte. d) Cycling stability of Zn/Ti asymmetric cell configuration in DMPU and aqueous electrolyte with corresponding CE values. Reproduced from ref.[29] Copyright (2024), with permission from the publisher. e) Schematic of AV‐based electrolyte fabrication steps. f) Electrochemical stability test of symmetric Zn//Zn cells in 2.0 m ZnSO_4_ AV‐based and aqueous electrolytes at a current density of 2.0 mA cm^−2^ (2.0 mAh cm^−2^). g) Ex situ XRD patterns of Zn anodes and h) Tafel plots in AV‐based and aqueous electrolytes. h) Schematics of Zn plating on Zn anodes in aqueous and AV‐based electrolytes. Reproduced from ref.[2] Copyright (2024), with permission from the publisher.

- **Ethylene glycol, Glycerol, and Polyols**: Hydroxyl (‐OH)groups in ethylene glycol, glycerol, and polyols (compounds containing multiple ‐OH groups) are incorporated into an aqueous electrolyte to modify the hydrogen bonding interactions among water molecules. Ethylene glycol has been used as a co‐solvent in hybrid electrolyte systems in ZIBs^[^ ^36^ ^]^ and Zn/S batteries (**Figure** **3**a).^[^ ^37^ ^]^ Due to its high viscosity and excellent solvation properties, glycerol can provide a more controlled environment for zinc‐ion transport, reducing the likelihood of dendrite formation (Figure  3b).^[^ ^38^ ^]^ The presence of a high surface charge density in polyhydroxylated molecules contributes to their outstanding zincophilicity and hydrophilicity. For example, trehalose, which is a naturally occuring disaccharide extensively utilized in the food industry for its functions as a moisturizer, preservative, and stabilizer, has been shown to be an effective electrolyte additive in ZnSO_4_‐based electrolyte for durable ZIBs. Polyhydroxy groups play a crucial role in the reconstruction of hydrogen‐bonded networks and reduce the reactivity of water molecules, thus mitigating the evolution of H_2_ gas. Additionally, the high concentration of polyhydroxy groups encourages the chemical adsorption of trehalose on the zinc surface, functioning as a protective interface that guards against corrosion.^[^ ^39^ ^]^ The introduction of the zincophilic polyol type surfactant, alkyl polyglycoside (APG), serves to promote the reorganization of the hydrogen bond network, thereby reducing the activity of free water. This alteration facilitates the transition of the zinc‐ion solvation structure from [Zn^2+^(H_2_O)_6_⋅SO_4_^2–^] (solvent‐separated ion pair, SSIP) to [Zn^2+^(H_2_O)_5_⋅OSO_3_^2–^] (contact ion pair, CIP), resulting in a decrease in the number of Zn^2+^‐solvated water molecules. Concurrently, the APG molecules preferentially adsorb onto the zinc surface, creating a dehydrated layer that effectively inhibits the HER activity and controls the diffusion of Zn^2+^ ions (Figure 3c).^[^ ^40^ ^]^
- **Bio‐ionic liquids**: Ionic liquids (ILs) represent a distinctive category of non‐volatile salts characterized by a range of advantageous properties. These include significant thermal stability, minimal volatility, an extensive electrochemical stability window, and adjustable physicochemical characteristics.^[^ ^14^ ^]^ Bio‐ionic liquids (ILs) such as choline acetate (ChAc) have also shown potential due to their non‐volatile and non‐flammable nature, which enhances the safety of Zn‐based energy storage systems (Figure 3d).^[^ ^41^, ^42^, ^43^ ^]^
- **Natural Deep Eutectic Solvents (DESs)**: One notable advancement involves the use of natural DES‐based electrolytes in ZIBs. DESs are mixtures of hydrogen bond donors (HBD) and acceptors (HBA) that form a eutectic system with a melting point lower than that of the individual components.^[^ ^44^ ^]^ Choline chloride is the most frequently employed HBA in the formulation of DES.^[^ ^45^ ^]^ Natural DESs are a specific class of eutectic solvents originating from natural materials, such as organic acids, alcohols, amino acids, sugars, and choline derivatives. These natural solvents have advantages, including affordability, sustainability, and biodegradability, which are particularly interesting for ZIBs due to their tunable properties, low toxicity, and ability to dissolve a wide range of salts.^[^ ^46^, ^47^ ^]^ Natural DES‐based electrolytes have demonstrated promising results in reducing HER and thus improving the cycling stability of ZIBs.^[^ ^48^, ^49^ ^]^ A study by Lin et al. (2024) showed that an acetamide‐based natural DES promoted the formation of a more uniform solid electrolyte interface (SEI) on the Zn anode, which suppresses the dendrites.^[^ ^47^ ^]^ In another study, Kao‐ian et al. formulated a natural DES made from ChCl and urea, proposing it as an alternative electrolyte for Zn‐ion batteries.^[^ ^50^ ^]^ These studies highlight the importance of natural DES for electrolyte design.
- **Biomass‐Derived Organic Solvents**: The reactions at the electrode–electrolyte interface can be efficiently controlled by formulating the electrolyte with biomass materials, which possess abundant functional groups and unique physicochemical properties. Functional groups such as amino, carboxyl, and hydroxyl groups form strong bonds with electrolyte components, and the organic moieties easily adjust ionic mobility and interfacial electric fields.^[^ ^32^ ^]^ Solvents derived from renewable biomass sources containing zincophilic O‐ and/or N‐ containing groups, such as lactones (GVL)^[^ ^30^ ^]^ and organic acids (phytic acid^[^ ^51^ ^]^ and 2‐aminoethanesulfonic acid,^[^ ^52^ ^]^ acetamide^[^ ^25^ ^]^ and urea^[^ ^28^ ^]^ (as part of water‐in‐DES electrolytes), offer another sustainable option for ZIB electrolytes. The research conducted by Wang et al. indicates that GVL can significantly decrease the desolvation energy of Zn^2+^ ions and enhance their transport kinetics. As a result, the electrolyte modified with 3% GVL exhibited outstanding reversible cycling performance at a current density of 1.0 mA cm^2^ and a capacity of 1.0 mAh cm^2^ in a Zn//Zn symmetric cell and improved the electrochemical stability of anodes (**Figure** **4**a,b).^[^ ^53^ ^]^ In another study, a green electrolyte utilizing N,N’‐Dimethylpropyleneurea (DMPU) as the sole solvent was utilized, resulting in a durable, dendrite‐free zinc anode demonstrating over 5000 h of cycling stability (Figure 4c,d).^[^ ^29^ ^]^ A recent investigation conducted by Chen et al. has revealed that natural biomass soups (NBSs), cost‐effective and readily available in nature, derived from reed biomass serve as innovative green solvents, offering a viable alternative to ILs and DESs in the recycling of LIB cathodes.^[^ ^26^ ^]^ A key benefit of NBSs is their ability to be utilized directly, eliminating the need for additional pretreatment and industrial separation or purification, often required for conventional electrolyte solvents. Solvents with strong hydrogen‐bonding capabilities tend to promote the formation of a more uniform and protective SEI, which can inhibit dendrite growth. Additionally, the SEI formed in natural solvent electrolytes can act as a physical barrier that limits the transport of zinc ions to specific sites on the anode surface, thereby promoting more uniform deposition. This helps to reduce the local current density and suppress the growth of dendrites, leading to improved battery performance and safety. Recently, our group reported that aloe vera (AV) can be used as a natural solvent with quinone‐rich organic molecules (Figure 4e).^[^ ^2^ ^]^ The rich organic moieties of the AV can significantly improve the Zn anode stability and enhance the anode stability over 4500 h (Figure 4f). This remarkable stability revealed that the interaction with organic molecules in AV alters the nucleation and diffusion dynamics of Zn^2+^, thereby promoting growth along the (002) crystal plane and preventing the formation of dendrites and side reactions (Figure 4g–i). This application of NBSs is particularly noteworthy due to their exceptional capacity to extract metals from waste cathodes at mild temperatures. It is encouraging that natural organic compounds in the NBSs demonstrate high efficiency in recovering metal ions via metal sequestering ability through complexation. This remarkable feature shows the potential of NBSs as natural solvents for high‐performance ZIBs.

## 4 Current Advances and Research Directions

Although there has been an acceleration in the field of ZIBs, the development of natural solvent‐based electrolytes for ZIBs is still in its infancy. Research has mainly focused on improving the electrolyte systems' electrochemical performance, stability, and sustainability. As discussed above, several studies have already demonstrated the potential of NADESs, biomass‐derived organic solvents, and bio‐ionic liquids in mitigating common challenges such as parasitic side reactions and dendrite formation (**Table** **1**). Despite the promising advances in natural solvent‐based electrolytes, several challenges must be overcome to enable their widespread adoption and the discovery of new formulations for practical applications such as grid‐scale and stationary energy storage systems. For example, the high viscosity of certain natural solvents, such as glycerol and DES, can be beneficial for controlling Zn^2+^ transport and safety. On the other hand, it can reduce the ionic conductivity, leading to slower charge/discharge rates. In addition to advances in green electrolyte formulations, specific improvements have been made in the Zn anode surface modifications.^[^ ^22^, ^54^ ^]^ For instance, a recent study utilized a protective layer composed of a carbon material that prevents zinc corrosion and improves cycling stability.^[^ ^22^ ^]^ However, the interactions between the Zn anode and the natural solvent‐based electrolytes must be enhanced to ensure that they can meet the performance demands of high‐power applications.

**Table 1:** Electrochemical stabilities with natural solvent‐based electrolytes.

Solvent	Salt	Cycling condition	Lifespan	Refs.
EG + H_2_O	Zn(OTf)_2_	1.0 mA cm^−2^, 1.0 mAh cm^−2^	4500 h	[46]
Aloe Vera	2 m ZnSO_4_	2.0 mA cm^−2^, 2.0 mAh cm^−2^	4600 h	[2]
HA (6.0 wt.%) + H_2_O	2 m ZnSO_4_	1.0 mA cm^−2^, 1.0 mAh cm^−2^	5500 h	[13]
GA + H_2_O	1 m ZnSO_4_	0.2 mA cm^−2^, 0.2 mAh cm^−2^	1300 h	[18]
15 *m* ChCl + H_2_O	30 *m* ZnCl_2_	0.2 mA cm^−2^	1600 h	[49]
0.5 m APG + H_2_O	2 m ZnSO_4_	1.0 mA cm^−2^, 1.0 mAh cm^−2^	5250 h	[40]
DMPU + H_2_O	0.5 m Zn(OTf)_2_	1.0 mA cm^−2^, 0.25 mAh cm^−2^	5000 h	[29]
[Ch]OAc + 30 wt.% H_2_O	1.0 m Zn(OAc)_2_	0.2 mA cm^−2^	200 h	[41]
GVL + H_2_O	ZnCl_2_	1.0 mA cm^−2^, 2.0 mAh cm^−2^	1100 h	[30]
0.25 mm PA + H_2_O	1.0 m ZnSO_4_	2.0 mA cm^−2^, 1.0 mAh cm^−2^	1000 h	[12]
EG: ChCl 4:1 (molar ratio)	1.0 m ZnSO_4_	0.5 mA cm^−2^, 0.2 mAh cm^−2^	800 h	[73]

Ethylene glycol (EG), Hyaluronic acid (HA), Gum arabic (GA), Choline chloride (ChCl), alkyl polyglycoside (APG) N,N’‐Dimethylpropyleneurea (DMPU), Choline acetate ([Ch]OAc), γ‐valerolactone (GVL), Phytic acid (PA).

Another promising approach is the development of electrolyte additives designed to enhance Zn^2+^ ion transport and control the deposition process.^[^ ^55^, ^56^ ^]^ Green and non‐toxic additives such as natural organic compounds, for example, sorbitol, xylose, trehalose, and APG have been shown to influence the ionic environment within the electrolyte, promoting more uniform deposition and reducing the likelihood of dendrite formation.^[^ ^57^, ^58^, ^59^, ^60^ ^]^ The abundant polar ‐OH groups within these molecules can act as hydrogen bond donors, influencing the behavior of free water and imparting pronounced zincophilic characteristics that facilitate the formation of a solvation structure for Zn^2+^ that is deficient in H_2_O. These electrolyte engineering techniques, when combined with natural solvent‐based electrolytes, have shown the potential to improve the performance and longevity of ZIBs significantly. Another challenge is the cost and scalability of natural solvent‐based electrolytes. While biomass‐derived solvents and NADESs are considered more sustainable than conventional organic solvents, large‐scale production of these materials may still be expensive.^[^ ^61^, ^62^ ^]^ For example, the use of bio‐ionic liquids, while offering excellent performance, is often cost‐prohibitive, which could limit their use in commercial ZIBs. Last, the integration of natural solvent‐based electrolytes into existing battery manufacturing processes poses technical challenges. These electrolytes often have different chemical and physical properties compared to aqueous electrolytes, requiring adjustments to electrode materials, separators, and cell designs. The successful application of these electrolyte systems necessitates comprehensive characterization and assessment to gain insight into their properties. For example, in operando optical microscopy can be used to investigate the corrosion mechanism on the Zn anode.^[^ ^63^ ^]^ Also, the characterization of Zn deposits and SEI layers after electrochemical cycling can be performed by X‐ray photoelectron spectroscopy.^[^ ^64^ ^]^

It is also essential for the practical implementation of ZIBs to develop solutions for a range of temperature conditions, both low and high, with the electrolyte being pivotal in attaining optimal performance. Lowering the freezing point of the electrolyte and preventing the formation of ice crystals are widely recognized as essential conditions for the effective operation of ZIBs in low‐temperature environments.^[^ ^65^ ^]^ In addition, elevating the operational temperature to a higher temperature (40–150 °C) can enhance the kinetics of reactions and facilitate ion transfer within a specific range. High‐temperature operations introduce critical challenges, including electrolyte volatility, side reactions, degradation of interfaces, self‐heating, and self‐discharge, all of which adversely affect the longevity and functionality of the device. Upon returning to ambient temperature, devices subjected to high‐temperature conditions may experience irreversible failure. Consequently, advancements and innovations in electrolytes designed for high‐temperature environments are crucial for applications that require a wide range of operating temperatures.^[^ ^66^ ^]^ To alleviate the issue of electrolyte volatility, natural solvents can increase the boiling point and decrease the water or liquid content in the electrolyte. Furthermore, controlling side reactions is essential, which can be achieved by ensuring a more stable electrochemical window and improving selectivity.^[^ ^67^ ^]^ The incorporation of EG, which can be derived from biomass, enhances the antifreeze properties of the electrolyte while also boosting its conductivity at lower temperatures.^[^ ^68^ ^]^ The robust hydrogen bonds established due to glycerol (or EG) significantly lower the freezing point and maintain elevated conductivity at subzero temperatures, thus providing high energy and power densities and long cycle life.^[^ ^69^, ^70^ ^]^ Incorporating non‐flammable and thermally stable natural solvents into the electrolyte is an effective strategy for the thermal stability of ZIBs.^[^ ^71^, ^72^ ^]^ Overcoming these challenges will require further research and innovation in material science and manufacturing techniques.

## 5 Summary and Outlook

Future research should focus on exploring new natural solvents, optimizing performance, improving long‐term cycling stability, integrating advanced electrode materials, and addressing environmental and economic sustainability. While some studies have demonstrated promising results, achieving long‐term stability and high‐performance cycling remains a challenge. This point is vitally essential for large‐scale energy storage applications, where reliability and longevity are critical. While significant advancements have been made in the laboratory conditions, translating these findings to practical, commercial‐scale applications will require further optimization. In this regard, future work should also focus on testing these systems under real‐world conditions and exploring their commercial potential in applications such as grid storage and electric vehicles. Addressing the challenges of viscosity and cost will also be essential for scaling these materials for industrial use. Finally, life cycle assessments should be conducted to evaluate the environmental footprint of different electrolyte formulations. Future work should focus on developing more efficient and less resource‐intensive methods for producing these solvents, as well as exploring recycling, reuse, and circular economy strategies to further reduce their environmental footprint. By overcoming these challenges, natural solvent‐based electrolytes can pave the way for the development of safer, more efficient, and more sustainable energy storage solutions, particularly in the growing field of ZIBs.

## Conflict of Interest

The authors declare no conflict of interest.

## Data Availability

Data sharing is not applicable to this article as no new data were created or analyzed in this study.
